# In vitro and in vivo reduction of post-prandial blood glucose levels by ethyl alcohol and water *Zingiber mioga* extracts through the inhibition of carbohydrate hydrolyzing enzymes

**DOI:** 10.1186/s12906-016-1090-4

**Published:** 2016-03-31

**Authors:** Sung-Hoon Jo, Cha-Young Cho, Jung-Yoon Lee, Kyoung-Soo Ha, Young-In Kwon, Emmanouil Apostolidis

**Affiliations:** Department of Chemistry and Food Science, Framingham State University, Framingham, MA 01701 USA; Department of Food and Nutrition, Hannam University, Daejeon, 305-811 South Korea

**Keywords:** α-glucosidase, Sucrase, Anti-hyperglycemia, Blood glucose, Oxygen radical absorbance capacity, *Zingiber mioga*

## Abstract

**Background:**

Type 2 diabetes is a serious problem for developed and developing countries. Prevention of prediabetes progression to type 2 diabetes with the use of natural products appears to be a cost-effective solution. *Zingiber mioga* has been used as a traditional food in Asia. Recent research has reported the potential health benefits of *Zingiber mioga*, but the blood glucose reducing effect has not been yet evaluated.

**Methods:**

In this study *Zingiber mioga* extracts (water and ethanol) were investigated for their anti-hyperglycemic and antioxidant potential using both in vitro and animal models. The in vitro study evaluated the total phenolic content, the oxygen radical absorbance capacity (ORAC) and the inhibitory effect against carbohydrate hydrolyzing enzymes (porcine pancreatic α-amylase and rat intestinal sucrase and maltase) of both *Zingiber mioga* extracts. Also, the extracts were evaluated for their in vivo post-prandial blood glucose reducing effect using SD rat and *db/db* mice models.

**Results:**

Our findings suggest that the ethanol extract of *Zingiber mioga* (ZME) exhibited the higher sucrase and maltase inhibitory activity (IC_50_, 3.50 and 3.13 mg/mL) and moderate α-amylase inhibitory activity (IC50, >10 mg/mL). Additionally, ZME exhibited potent peroxyl radical scavenging linked antioxidant activity (0.53/TE 1 μM). The in vivo study using SD rat and *db/db* mice models also showed that ZME reduces postprandial increases of blood glucose level after an oral administration of sucrose by possibly acting as an intestinal α-glucosidase inhibitor (ZME 0.1 g/kg 55.61 ± 13.24 mg/dL)

**Conclusion:**

The results indicate that *Zingiber mioga* extracts exhibited significant in vitro α-glucosidase inhibition and antioxidant activity. Additionally, the tested extracts demonstrated in vivo anti-hyperglycemic effects using SD rat and *db/db* mice models. Our findings provide a strong rationale for the further evaluation of *Zingiber mioga* for the potential to contribute as a useful dietary strategy to manage postprandial hyperglycemia.

## Background

Non-insulin dependent diabetes mellitus (NIDDM, Type 2 diabetes) is a group of metabolic diseases marked by high levels of blood glucose resulting from faults in insulin resistance and insulin production [1–3]. Hyperglycemia, a rapid rise in blood glucose levels in NIDDM patients occurs due to hydrolysis of starch by pancreatic α-amylase and absorption of glucose in the small intestine by α-glucosidases such as sucrase and maltase [3]. Inhibition of these carbohydrate-hydrolyzing enzymes can significantly decrease the postprandial hyperglycemia after a mixed carbohydrate diet and can be a key strategy in the control of diabetes mellitus [4, 5]. However, a main negative aspect of currently used therapeutic α-glucosidase inhibitors, such as the drug acarbose, is the strong α-amylase inhibitory activity causing abnormal bacterial fermentation of undigested starch in the colon that leads to abdominal distention, flatulence, meteorism and possibly diarrhea [6, 7]. Recent studies showed that phenolic phytochemicals from plant sources are natural inhibitors of α-amylase and α-glucosidase [8, 9] Such natural inhibitors from dietary plants could be useful since they have lower inhibitory activity against α-amylase and a stronger inhibitory activity against α-glucosidase and can be potentially used as an effective strategy for the management of postprandial hyperglycemia with minimal side effects [9].

*Zingiber mioga* (Zingiberaceae family) is a herb original to eastern Asia. Its young flower buds have been used as a traditional food in Asia [10]. Recent research has reported the antimicrobial activities of the constituents of *Zingiber mioga* against several strains of bacteria, yeast, and mold [11, 12]. The volatile ingredients of *Zingiber mioga* have been studied; 2-isopropyl-3-methoxypyrazine, 2-sec-butyl-3-methoxypyrazine, and 2-isobutyl-3-methoxypyrazine were found to be the aroma compounds by GC-MS [1]. Additionally, mioga extract was effective in inhibiting fat accumulation in 3 T3-L1 adipocytes leading to a decrease in body weight gain and a decrease in fat mass in ICR mice [2]. However, the significance of *Zingiber mioga* intake for preventing diabetes-related oxidative stress and hyperglycemia is not reported. Based on the previous obesity related findings, it is interesting to first evaluate the in vitro potential of mioga extracts again carbohydrate hydrolyzing enzyme using in vitro models and if inhibitory effect is observed, then the extracts should be evaluated using an animal model.

Therefore, the aim of this study is to examine the potential effect and mechanism of action of *Zingiber mioga* extract on the inhibition of postprandial hyperglycemia using both in vitro and in vivo animal models. Clear knowledge of the activity and mode of action of *Zingiber mioga* extract will contribute towards better understanding of the actual effect of various *Zingiber mioga* products towards type 2 diabetes management. To determine the above, in this study, we (i) prepared *Zingiber mioga* extracts (water extract of *Zingiber mioga*: ZMW, ethanol extract of *Zingiber mioga*: ZME); (ii) investigated the inhibitory activity of ZMW, ZME against α-amylase and α-glucosidase (anti-hyperglycemia potential); (iii) measured antioxidant potential using oxygen radical scavenging capacity (ORAC) assay; and (iv) evaluated the postprandial blood glucose lowering effect of ZMW, ZME after sucrose loading in a Sprague-Dawley (SD) rat and db/db mice model.

## Methods

### Chemicals

*Zingiber mioga* was obtained from a local market in Jeju, Korea. The purchased samples were identified by one of the authors (Young-In Kwon). A voucher specimen (BFC O10985) was deposited at the Bioactive Food Components Lab (BFCL) of the College of Life Science and Nano Technology, Hannam University. Rat intestinal acetone powder, porcine pancreatic α-amylase enzyme powder, starch, sucrose, and maltose were purchased from Sigma-aldrich (St. Luis, MO, USA). Unless noted, all chemicals were purchased from Sigma-aldrich (St. Luis, MO, USA).

## Preparation of *Zingiber mioga* extracts

### Water extraction

*Zingiber mioga* was crushed to a fine powder and was extracted by autoclaving the ground leaves at 121 °C for 15 min with one gram-fresh weight per 40 mL of distilled water. *Zingiber mioga* extract was then centrifuged at 8000 x g for 30 min, filtered through a Whatman # 1 filter, vacuum-evaporated at 60 °C, freeze dried and kept at -20 °C refrigerator until analysis.

### Ethanol extraction

*Zingiber mioga* was crushed to a fine powder and was extracted by Shaking Incubation at 40 °C for 2 h with one gram-fresh weight per 40 mL of ethanol. *Zingiber mioga* extract was then centrifuged at 8000 x g for 30 min, filtered through a Whatman # 1 filter, vacuum-evaporated at 60 °C, freeze dried and kept at -20 °C refrigerator until analysis.

## Carbohydrate hydrolyzing enzyme inhibition

### Porcine pancreatic α–amylase inhibition assay

Porcine pancreatic α-amylase inhibition was determined by the method described by Kwon et al [3]. Sample solution (200 μL) and 0.02 M sodium phosphate buffer (pH 6.9 with 0.006 M sodium chloride, 500 μL) containing α-amylase solution (0.5 mg/mL, 5.0 MU/mL) were incubated at 25 °C for 10 min. After pre-incubation, 500 μL of a 1 % starch solution in 0.02 M sodium phosphate buffer was added. The reaction mixture was then incubated at 25 °C for 10 min. The reaction was stopped with 1.0 mL of dinitrosalicylic acid (DNS). The reaction mixture was then incubated in a boiling water bath for 5 min and cooled to room temperature. The reaction mixture was then diluted after adding distilled water, and absorbance was measured at 540 nm with ELISA microplate reader (SUNRISE; Tecan Trading AG, Saltzburg, Austria).

$$ \%\ \mathrm{inhibition} = \left(\left[\frac{\Delta {A}_{540}^{Control}-\Delta {A}_{540}^{Extract}}{\left[\Delta {A}_{540}^{Control}\right]}\right]\right)x100 $$

### Sucrase and Maltase inhibition assay

The crude enzyme solution prepared from rat intestinal acetone powder Sigma-Aldrich Co. (St. Louis, MO, USA) was used as the small intestinal maltase, sucrase, and glucoamylase, showing specific activities of 0.70, 0.34 and 0.45 units/mL, respectively. Rat intestinal acetone powder (1.0 g) was suspended in 3 mL of 0.9 % saline, and the suspension was sonicated twelve times for 30 s at 4 °C. After centrifugation (10,000 × g, 30 min, 4 °C), the resulting supernatant was used for the assay. Sucrase and maltase inhibitory activity were assayed by modifying a method developed by Dahlqvist (1964) [4]. The inhibitory activity was determined by incubating a solution of an enzyme (50 μL), 0.1 M phosphate buffer (pH 7.0, 100 μL) containing 0.4 mg/mL sucrose or maltose or 1 % soluble starch, and a solution (50 μL) with various concentrations of sample solution (between 0.05 mM and 1.0 mM) at 37 °C for 30 min. The reaction mixture was heated in a boiling water bath to stop the reaction for 10 min, and then the amount of liberated glucose was measured by the glucose oxidase method [5]. The inhibitory activity was calculated from the formula as follows. Inhibition (%) = (C-T)/C x 100, where C is the enzyme activity without inhibitor and T is the enzyme activity with inhibitor.

### Oxygen radical absorbance capacity (ORAC) assay

The peroxyl radical-scavenging capacities of *Zingiber mioga* extracts were measured using ORAC [6, 7]. The ORAC assay was carried out using a Tecan GENios multi-functional plate reader (GENios; Tecan Trading AG, Salzburg, Austria) with fluorescent filters (excitation wavelength: 485 nm, emission filter: 535 nm). In the final assay mixture, fluorescein (40 nM) was used as a target of free radical attack with either 2, 2′-azobis (2-amidinopropane) dihydrochloride (AAPH, 20 mM) as a peroxyl radical generator in peroxyl radical-scavenging capacity (ORAC_ROO·_) assay or with H_2_O_2_ - CuSO_4_ (H_2_O_2_, 0.75 %; CuSO_4_, 5 μM) as a hydroxyl radical generator in hydroxyl radical-scavenging capacity (ORAC_HO·_) assay. Trolox (1 μM) was used as a control standard and prepared fresh on a daily basis. The analyzer was programmed to record the fluorescence of fluorescein every 2 min after AAPH or H_2_O_2_ - CuSO_4_ was added. All fluorescence measurements were expressed relative to the initial reading. Final results were calculated based on the difference in the area under the fluorescence decay curve between the blank and each sample. All data were expressed as micromoles of Trolox equivalents (TE). One ORAC unit is equivalent to the net protection area provided by 1 μM of Trolox.

### Total phenolic content assay

The total phenolic content was determined by an assay modified from Velioglu et al. [8, 9]. One milliter of sample solution was transferred into a test tube and mixed with 1 mL of 95 % ethanol and 5 mL of distilled water. To each sample 0.5 mL of 50 % (v/v) Folin-ciocalteu reagent was added and mixed. After 5 min, 1 mL of 5 % Na_2_CO_3_ was added to the reaction mixture and allowed to stand for 1 h. The absorbance was read at 725 nm using spectrophotometer (UV-160A; Shimadzu Inc., Kyoto, Japan). The absorbance values were converted to total phenolics and were expressed in mg equivalents of gallic acid/mL of the sample. Standard curves were established using various concentrations of gallic acid in 95 % ethanol.

### Animal and study design

The inhibitory effect of *Zingiber mioga* extacts and acarbose on postprandial hyperglycemia after carbohydrate loads in Sprague-Dawley (SD) rats and C57BL/KsJ-*db*/*db* (*db*/*db*) mice were evaluated. The experimental protocols followed were approved by the Institutional Animal Care and Use Committee (IACUC) of the Hannam University (Approval number: HNU2015-0004). Five week-old male SD rats and C57BL/KsJ-*db*/*db* (*db*/*db*) mice were purchased from Joongang Experimental Animal Co. (Seoul, Korea) and fed a solid diet (Samyang Diet Co., Seoul, Korea) for 1 week. The rats and mice were housed in a ventilated room at 25 ± 2 °C with 50 ± 7 % relative humidity, and under an alternating 12 h light/dark cycle. After 4 groups of 5 male SD rats and *db/db* mice were fasted for 24 h, 2.0 g/kg of sucrose were orally administrated concurrently with 0 ~ 100 mg/kg inhibitors (*Zingiber mioga extracts* or Acarbose). The blood samples were then taken from the tail after administration and blood glucose levels were measured at 0, 0.5, 1, and 2 h. The glucose level in blood was determined by glucose oxidase method and compared with that of the control group, which had not taken the inhibitors.

### Statistical analysis

All data are presented as mean ± Standard deviation (SD). Statistical analysis was carried out using statistical package SPSS 10 (Statistical Package for Social Science; SPSS Inc., Chicago, IL, USA) program and significance of each group was verified with the analysis of on-way analysis of variance (ANOVA) followed by the Duncan’s test of *p* < 0.05.

## Results and discussion

### Total phenolic contents in *Zingiber mioga* extracts

The total phenolic content in the two *Zingiber mioga e*xtracts (ZME; Ethyl alcohol extract of *Zingiber mioga*, ZMW; Water extract of *Zingiber mioga*) was evaluated. We determined that ZME had a total phenolic content of 2697.31 mg/100 g which was higher than ZMW (2182.96 mg/100 g) (Table 1). These results suggest that ethanol is a more efficient solvent for phenolic compounds extraction compared to water. Previous reports have shown that the total polyphenol contents in extracts has a positive correlation with observed anti-hyperglycemic activity in diabetic rats [11]. Other studies using specific phenolic compound standards such as caffeic acid, catechins, anthocyanins, quercetins, and their derivatives confirmed their hypoglycemic effects in vitro and in vivo [12].

**Table 1:** Total phenolci content of *Zingiber mioga* extracts

	Total phenolic contents (mg/100 g)
ZMW	2182.96 ± 48.02
ZME	2697.31 ± 118.25

ZMW: Water extract of *Zingiber mioga*

ZME: Ethanol extract of *Zingiber mioga*

### In vitro antioxidant capacity: peroxyl and hydroxyl radical scavenging capacity

**Fig. 1:**
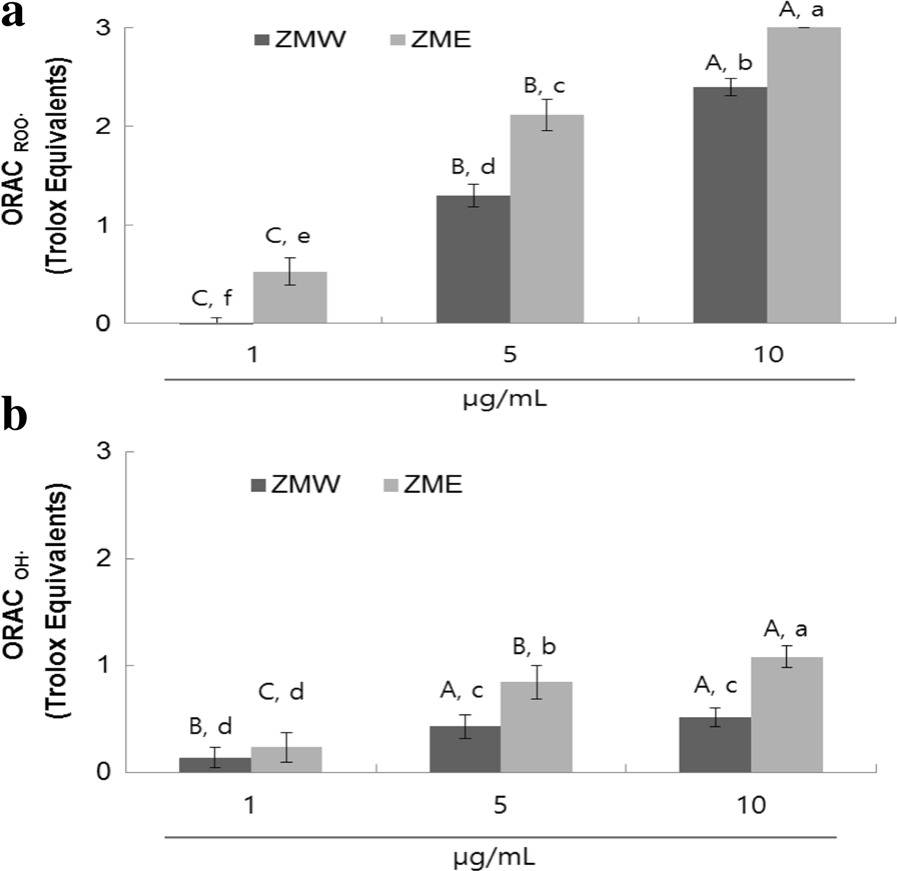
Dose dependent changes in peroxyl and hydroxyl radical scavenging activities (Trolox equivalent, μM) of ZMW and ZME. **a** Peroxyl radical scavenging activity of extracts; **b** hydroxyl radical scavenging activity of extracts. The oxygen radical absorbance capacity (ORAC) value is calculated by dividing the area under the sample curve by the area under the Trolox curve, with both areas being corrected by subtracting the area under the blank curve. One ORAC unit is assigned as the net area of protection provided by Trolox at a final concentration of 1 μM. The area under the curve for the sample is compared to the area under the curve for Trolox, and the anti-oxidative value is expressed in micromoles of Trolox equivalent per liter. The results represent the mean ± SD. of values obtained from three measurements. Different corresponding letters indicate significant differences at *p* < 0.05 by Duncan’s test

The antioxidant activity of *Zingiber mioga* extracts was investigated for their radical-scavenging capacity using the ORAC assay system. Figure 1 demonstrates that the scavenging activity of *Zingiber mioga* extracts on peroxyl radicals generated from AAPH is dose-dependent at doses ranging from 1 to 10 μg/mL. The bars in Fig. 1a represent the ORAC _ROO·_ activity of 1 μM of the tested sample equivalent to 1 μM Trolox, a water-soluble α-tocopherol analogue. The ORAC value for the sample extracts ranged from 0.53 ± 0.14 to 3.10 ± 0.10 μM of TE. At all tested doses, ZME exhibited higher scavenging activity on peroxyl radicals generated from AAPH compared to ZMW (Fig. 1a). Furthermore, we evaluated the hydroxyl radical absorbing activity (ORAC _OH·_) of ZME and ZMW using ORAC assay in which Cu^2+^ and H_2_O_2_ were used as hydroxyl radical generator. Similarly to our previous findings, ZME had higher scavenging activity compared to ZMW (Fig. 1b). Oxygen Radical Absorbance Capacity (ORAC) assay system has been used successfully to determine the reaction capacity with peroxyl radical, one of the harmful and reactive oxygen species in biological. ORAC assay has been employed widely in testing foods and beverages to provide an integrated and quantitative determination of “total antioxidant capacity” (TAC). The ORAC value is obtained by measuring the area under the curve (AUC) of the magnitude and time of inhibition of free radical attack on a fluorescent target molecule in reference to Trolox (6-hydroxy-2,5,7,8-tetramethylchroman-2-carbonyl acid) as a unit standard. Our findings suggest that the ethyl alcohol extract has higher antioxidant potential than the water extract. Phenolic compounds are well-documented radical scavengers and our findings (Fig. 1) correlate with the observed total phenolic contents (Table 1).

### Sucrase and maltase inhibition

**Fig. 2:**
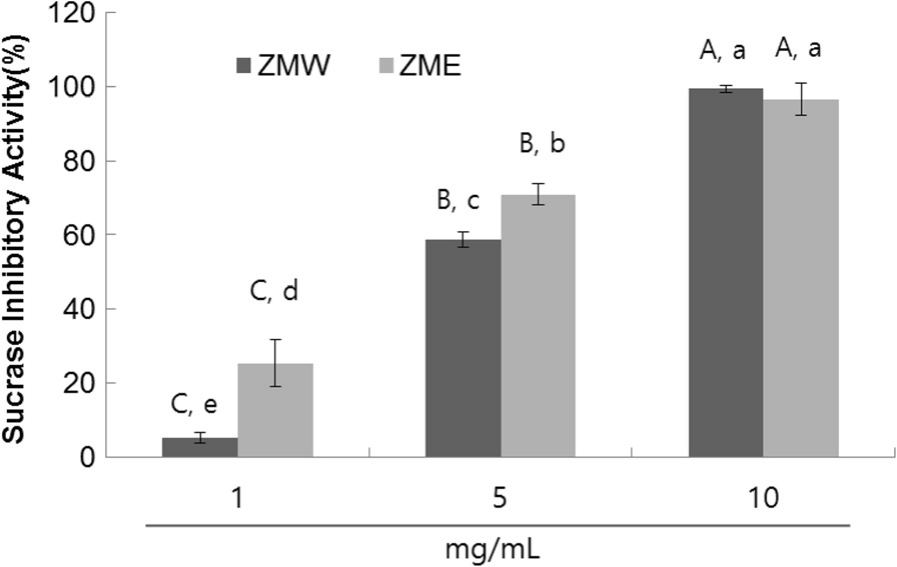
Dose-dependent changes in sucrase inhibitory activities (% inhibition) of ZMW and ZME. The results represent the mean ± S.D. of values obtained from 3 measurements. Different corresponding letters indicate significant differences at *P* < 0.05 by Duncan’s test. First letter is among different samples and second one is among different concentrations within same samples

**Fig. 3:**
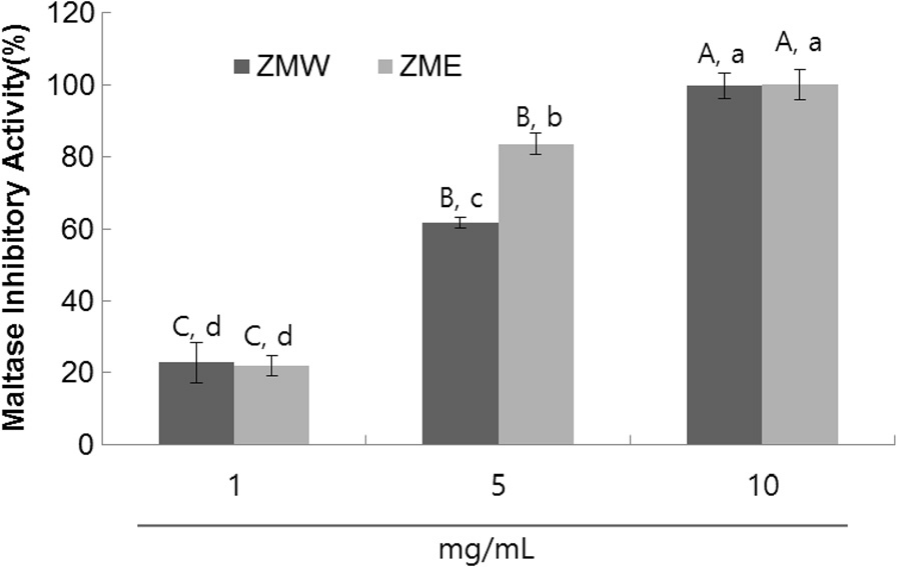
Dose-dependent changes in maltase inhibitory activities (% inhibition) of ZMW and ZME. The results represent the mean ± S.D. of values obtained from 3 measurements. Different corresponding letters indicate significant differences at *P* < 0.05 by Duncan’s test. First letter is among different samples and second one is among different concentrations within same samples

The dose-dependent sucrase inhibitory activities of ZME and ZMW were evaluated as outlined in materials and methods. The results showed that ZME exhibited significantly higher sucrase inhibitory activities (Fig. 2). More specifically, ZME had an IC_50_ value of 3.50 mg/mL, while ZMW had 4.91 mg/mL (Table 2). When maltase inhibitory activities were evaluated, similarly to sucrase inhibitory activity, ZME had higher maltase inhibitory activity (Fig. 3). More specifically, ZME and ZMW had IC_50_ values of 3.13 mg/mL and 3.99 mg/mL, repsectively (Table 2).

**Table 2:** Sucrase and maltase IC_50_ values of *Zingiber mioga* extracts

	Sucrase (mg/mL)	Maltase (mg/mL)
ZMW	4.91	3.99
ZME	3.50	3.13

ZMW: Water extract of *Zingiber mioga*

ZME: Ethanol extract of *Zingiber mioga*

The enterocyte of the small intestine can only absorb monosaccharaides such as glucose and fructose from our diet. The intestinal absorption of dietary carbohydrates such as maltose and sucrose is supported by a group of α-glucosidases, which include intestinal maltase, sucrase, glucoamylase and isomaltase. Inhibition of these enzymes can significantly decrease the postprandial increase of blood glucose level after a mixed carbohydrate diet [10]. Our results suggest that both extracts resulted to significant inhibition of the evaluated carbohydrate hydrolyzing enzymes. More specifically, when looking at the dose-dependent inhibitory activity, we can suggest that ZME had a higher inhibitory effect at the 5 mg/mL tested dose, which also results to a lower IC_50_ value. This observation could be due to the fact that ethanol extraction results to higher phenolic content when compared to the water extraction. Previous reports have clearly documented that carbohydrate hydrolyzing enzyme inhibitory activity depends on phenolic content [13].

### α-Amylase Inhibition of *Zingiber mioga* extracts

**Fig. 4:**
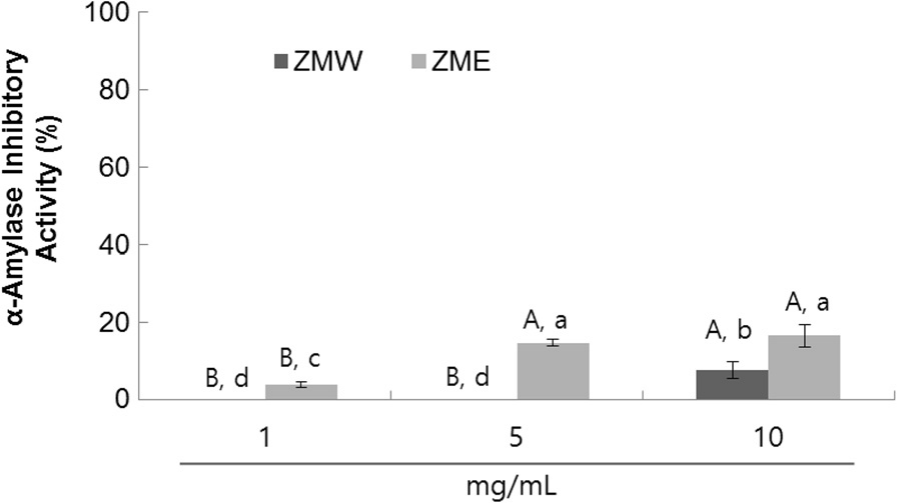
Dose-dependent changes in porcine pancreatic α-amylase inhibitory activities (% inhibition) of ZMW and ZME. The results represent the mean ± S.D. of values obtained from 3 measurements. Different corresponding letters indicate significant differences at *P* < 0.05 by Duncan’s test. First letter is among different samples and second one is among different concentrations within same samples'

The dose-dependent inhibitory activity of the *Zingiber mioga* extracts against porcine α-amylase was evaluated in this study. As seen in Fig. 4, both samples exhibited low α-amylase inhibitory activity, however ZME had a higher α-amylase inhibitory activity (16.44 ± 2.95 %) than ZMW (7.58 ± 2.28 %), (Fig. 1) at the same concentration (10 mg/mL).

α-Amylase inhibitors could slow the liberation of maltose from starch, resulting in delaying maltose conversion to glucose and decreasing postprandial plasma glucose levels. Previous reports with phenolic enriched herbal extracts reported a link between α-amylase and α-glucosidase inhibitory activity [3, 14]. All the herb extracts showed a comparable inhibition of α-glucosidase but did not have any inhibitory activity against porcine pancreatic α-amylase. Our findings are consistent with previous reports, since we observed significantly lower α-amylase inhibitory activity, compared to the sucrose and maltase inhibitory activities.

### Sucrose loading test in SD Rat and *db/db* mice model

**Fig. 5:**
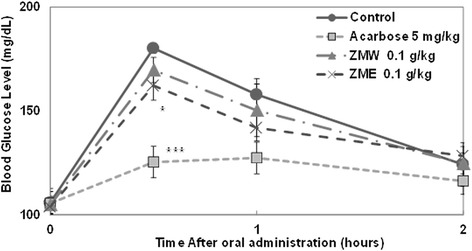
Effect of ZMW and ZME on sucrose loading test. After fasting for 24 h, 6-week-old, male SD rats were orally administered with sucrose solution (2.0 g/kg) with or without samples. Each point represents mean ± SD. (*n* = 5). *p < 0.05, **p < 0.01, and ***p < 0.001 compared to different samples at the same concentration by unpaired Student’s t-test

To confirm the in vivo relevance of our in vitro findings that ZME and ZMW extracts exhibited α-glucosidases inhibitory activities, we performed a sucrose loading test in SD rat, which is a model more relevant towards type 2 diabetes prevention with normal or pre-diabetic individuals, rather than type 2 diabetes treatments. In SD rats, ZME (0.1 g/kg) exerted a statistically significant decrease (*p* < 0.05) in the blood glucose at half an hour after sucrose loading. ZME significantly reduced (*p* < 0.05) the postprandial hyperglycemia caused by sucrose loading to an extent less than that observed in the acarbose (0.005 g/kg) administered group (*p* < 0.001) (Fig. 5). However, ZMW had no significant effect (Fig. 5).

**Fig. 6:**
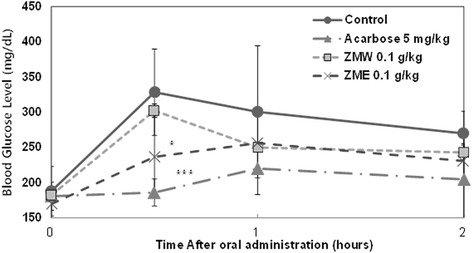
Effect of ZMW and ZME on sucrose loading test. After fasting for 24 h, 12-week-old, male *db/db* mice were orally administered with sucrose solution (2.0 g/kg) with or without samples. Each point represents mean ± SD. (*n* = 5). *p < 0.05, **p < 0.01, and ***p < 0.001 compared to different samples at the same concentration by unpaired Student’s t-test

Furthermore, we evaluated the effect of ZME and ZMW in 12 weeks *db/db* mouse model. Our observations suggested that ZME (0.1 g/kg) exerted a statistically significant decrease (*p* < 0.05) in the blood glucose at half an hour after sucrose loading (Fig. 6). Similarly to our previous observations using SD rat model, ZMW had no significant effect on the observed post-prandial blood glucose levels (Fig. 6).

Our results demonstrate the positive effects of ZME against post-prandial blood glucose levels resulting from high sucrose meal ingestion. Our in vivo findings, correlate with our in vitro observations, suggesting that ethanol alcohol extraction is more efficient than water extraction. More specifically, we have determined that ZME has higher phenolic content that results to a higher in vitro inhibition of carbohydrate hydrolyzing enzymes, when compared to ZMW. These observations translate to a higher in vivo efficacy of ZME.

## Conclusions

A sudden increase in blood glucose levels, causing hyperglycemia in NIDDM is due to insulin’s inability to effectively utilize the glucose produced by the hydrolysis of dietary carbohydrates by carbohydrate hydrolyzing enzymes. Furthermore, hyperglycemia-induced microvascular complications are likely from oxidative dysfunction from mitochondrial reactive oxygen species (ROS). Therefore, it is important to control both cellular redox status and blood glucose level for managing these diabetic complications. The ethanol extract of *Zingiber mioga* has α-glucosidase inhibitory activity and potent peroxyl radical scavenging-linked antioxidant activity. Sucrose loading test showed that ZME may reduce postprandial increases of blood glucose level by acting as an intestinal α-glucosidase inhibitor. The above dual benefits (post-prandial blood glucose reduction and antioxidant activity) of ZME could support the evidence that diet rich in fruits and vegetables are associated with lower incidences of oxidation-linked diseases such as diabetes [15–17]. Furthermore, the *Zingiber mioga* extracts showed a strong inhibition of α-glucosidase and low inhibition of α-amylase and therefore could be potentially used as an effective complementary therapy for postprandial hyperglycemia linked to type2 diabetes with reduced side effects [18]. It is well documented that the major side-effects of carbohydrate hydrolyzing enzyme inhibition are flatulence and diarrhea resulting from the high α-amylase inhibitory activity [19, 20].

These in vitro and in vivo studies provide the biochemical rationale for the potential benefit of *Zingiber mioga* for carbohydrate hydrolyzing enzyme inhibition, with ZME appearing to be more bioactive. This observed higher efficacy of ZME can be further enhanced by identifying the bioactive components responsible for the observed activity. Further investigation is underway to identify the specific phenolic compounds in *Zingiber mioga* extraction that are relevant to the inhibition of carbohydrate hydrolysis enzymes.

### Availability of data and materials

All data and materials are contained and described within the manuscript.
